# Fruquintinib Plus FOLFIRI/FOLFOX as Second‐Line Therapy in Patients With RAS‐Mutant Metastatic Colorectal Cancer: A Multicenter, Open‐Label, Single‐Arm Phase II Study

**DOI:** 10.1002/mco2.71137

**Published:** 2026-11-08

**Authors:** Yun Xu, Cong Li, Yiwei Zeng, Yuchen Wu, Yuedi Dai, Xiaopin Ji, Zhe Cui, Xiaodong Zhu, Ming Tang, Ye Xu

**Affiliations:** ^1^ Department of Colorectal Surgery Fudan University Shanghai Cancer Center Shanghai China; ^2^ Department of Oncology, Shanghai Medical College Fudan University Shanghai China; ^3^ Department of Oncology Shanghai Minhang Cancer Center Shanghai China; ^4^ Ruijin Hospital Shanghai Jiao Tong University School of Medicine Shanghai China; ^5^ Baoshan Branch of Renji Hospital Affiliated to Shanghai Jiao Tong University School of Medicine Shanghai China; ^6^ Shanghai Key Laboratory of Maternal Fetal Medicine, Shanghai Institute of Maternal‐Fetal Medicine and Gynecologic Oncology, Clinical and Translational Research Center, Shanghai First Maternity and Infant Hospital, School of Medicine Tongji University Shanghai China

**Keywords:** conversion surgery, fruquintinib, metastatic colorectal cancer, phase II trial, RAS‐mutant, second‐line therapy

## Abstract

For patients with RAS‐mutant metastatic colorectal cancer (mCRC), effective second‐line therapies remain limited. In this multicenter, single‐arm phase II trial conducted from March 2023 to November 2025, we enrolled 42 patients with RAS‐mutant mCRC who had progressed after first‐line therapy. All patients received fruquintinib (4 mg once daily on Days 1–21 of each 28‐day cycle) combined with either FOLFIRI or FOLFOX chemotherapy based on prior treatment history. The median progression‐free survival (PFS) was 8.08 months (95% CI: 6.18, 9.46), ranking among the longest reported in the second‐line setting. The objective response rate (ORR) was 52.5% (95% CI: 36.13, 68.49) and the disease control (DCR) rate 97.5% (95% CI: 86.84, 99.94). Notably, this study reports the first conversion surgery outcomes in this setting, with three patients (7.14%) undergoing successful resection—a rate approaching that observed in first‐line trials. The safety profile was manageable, with 7.1% of patients experiencing grade ≥ 3 adverse events including diarrhea (4.8%) and thrombocytopenia (2.4%); no treatment‐related deaths occurred. Prior bevacizumab treatment did not affect the consistent efficacy of this regimen. These results support further randomized evaluation of this fruquintinib‐based combination as a promising second‐line option for patients with RAS‐mutant mCRC.

## Graphical abstract

This phase II trial demonstrates that fruquintinib combined with chemotherapy achieves a median progression‐free survival of 8.08 months and a 52.5% objective response rate as second‐line therapy for RAS‐mutant metastatic colorectal cancer, with a notable 7.14% conversion surgery rate. The regimen shows consistent efficacy regardless of prior bevacizumab use and a manageable safety profile. These promising results support its further evaluation as a new second‐line therapeutic option for RAS‐mutant metastatic colorectal cancer.

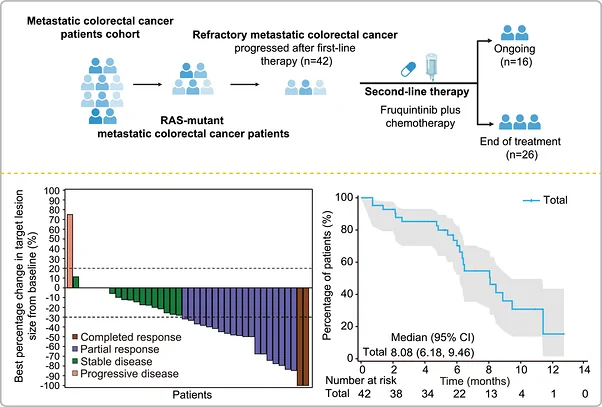

## 1 Introduction

Metastatic colorectal cancer (mCRC) poses a major global health challenge, being a key cause of cancer‐related mortality [1]. In China, its incidence and mortality are continuously increasing. Especially challenging aspect of this disease is that approximately 40% of patients present with de novo metastatic disease, for whom curative intervention is often not feasible, and treatment is primarily palliative [2]. Nearly half of all mCRC patients harbor RAS mutations, with KRAS and NRAS accounting for approximately 40% and 4% of cases, respectively, while HRAS mutations are rare (< 1%). Over 95% of these mutations cluster in three key hotspots–G12, G13, and Q61–which are critical for oncogenic activation [3].

The treatment landscape for mCRC has evolved from the LV/5‐FU era, which offered a median overall survival (OS) of only 8–12 months, to current combination chemotherapy (FOLFOX/FOLFIRI) and targeted agents against EGFR or VEGF, leading to further survival improvements [4, 5, 6, 7]. However, most patients progress following first‐line therapy. For the substantial subgroup with RAS mutations, anti‐EGFR therapy is contraindicated [8]. Further options are defined by other biomarkers, including immunotherapy for dMMR/MSI‐H tumors and later‐line regimens [9, 10]. Guided by the National Comprehensive Cancer Network Guidelines, the second‐line treatment for RAS‐mutant mCRC primarily involves combination chemotherapy (FOLFIRI or FOLFOX) together with the anti‐VEGF antibody bevacizumab [11].

Within this regimen, current guidelines support the “cross‐line” continuation of bevacizumab beyond first‐line progression. However, the long‐term survival benefit of this specific strategy remains uncertain. This is exemplified by the phase II TRUSTY study, which specifically assessed bevacizumab beyond progression and found no significant improvement in either progression‐free survival (PFS) or OS for patients [12]. Even in trials that reported improved PFS with continued bevacizumab, such as BEBYP and ML18147, the translation into a conclusive, meaningful OS benefit was inconsistent [13, 14]. Taken together with the neutral survival outcomes of these previous studies, cross‐line bevacizumab therapy may not reliably deliver substantial long‐term survival advantages, underscoring a clear unmet therapeutic need.

Given the limited long‐term survival with current strategies, interest has grown in VEGF‐targeting tyrosine kinase inhibitors (TKIs). Phase III trials such as CORRECT and RECOURSE validate this class in later‐line RAS‐mutant mCRC, reaffirming the importance of sustained VEGF pathway inhibition [15, 16, 17]. TKIs may overcome resistance via mechanisms distinct from monoclonal antibodies, prompting exploration of their earlier use [18, 19]. However, robust evidence directly comparing second‐line “cross‐line” bevacizumab versus a TKI switch remains lacking. At present, no studies on using TKIs as a second‐line therapy in mCRC have been reported. To address the current lack of clinical evidence, we performed this single‐arm phase II study to explore the efficacy and safety of a novel TKI plus chemotherapy regimen in this setting.

Fruquintinib (HMPL‐013), an oral, highly specific small‐molecule VEGFR‐1/2/3 inhibitor, represents a promising candidate for such an approach [20]. Its significant survival benefits in treatment‐refractory mCRC (demonstrated in the FRESCO and FRESCO‐2 phase III trials), along with a distinct mechanism that may overcome bevacizumab resistance, provided the rationale for its selection in our study [21, 22]. Given its proven efficacy, manageable safety profile, and the strong rationale for combining VEGF inhibition with chemotherapy [23, 24, 25], fruquintinib represents a compelling candidate for integration with standard regimens.

Therefore, this multicenter, open‐label, single‐arm phase II investigation assesses the activity and tolerability of fruquintinib plus FOLFIRI or FOLFOX chemotherapy for second‐line treatment of RAS‐mutant mCRC. The key findings include a median PFS of 8.08 months, an ORR of 52.5%, and a 7.14% conversion surgery rate, with a manageable safety profile. These results provide a promising second‐line option for this challenging patient population.

## 2 Results

### 2.1 Patient Characteristics

To characterize the study population, between March 15, 2023 and November 11, 2025, 47 patients were screened and 42 patients with mCRC were eligible and included in the study (Figure 1). All 42 participants received treatment and were evaluated for response and safety. Among the 42 enrolled patients, 38 were from Fudan University Shanghai Cancer Center, 3 from Shanghai Minhang Cancer Center, and 1 from Ruijin Hospital. Their ages ranged from 35 to 74 years (median 63.0 years), with 59.5% male and 40.5% female participants. The majority of patients had left‐sided primary tumors (73.8%), and 59.5% had liver metastases at baseline. Most patients had an ECOG performance status of 1 (66.7%), and 73.8% had previously received bevacizumab treatment. Most patients received FOLFIRI (*n* = 39, 92.9%), while 3 patients (7.1%) received FOLFOX. Table 1 summarizes the baseline demographic and clinical characteristics. Overall, the baseline characteristics were representative of a typical RAS‐mutant mCRC population.

**FIGURE 1:**
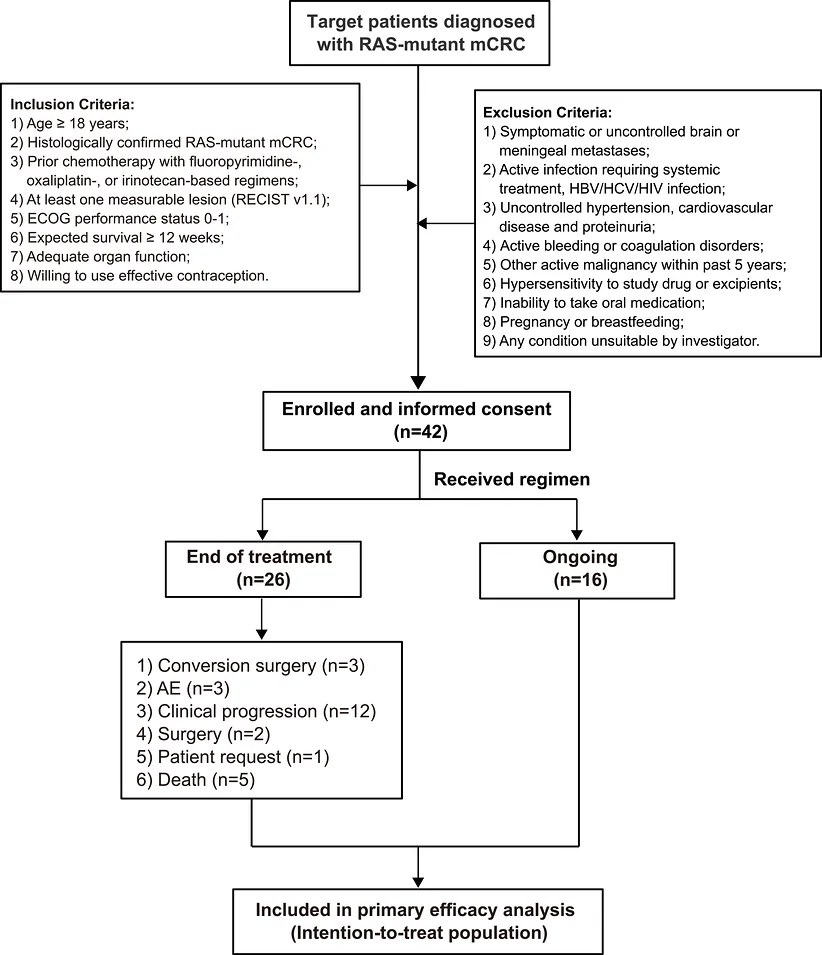
CONSORT flow diagram of trial participants.

**TABLE 1:** Baseline demographics and disease characteristics.

Characteristics	Patients (*n* = 42)
Median age, years	63.0 (35–74)
Gender, No. (%)	
Female	17 (40.5)
Male	25 (59.5)
ECOG PS, No. (%)	
0	14 (33.3)
1	28 (66.7)
Primary tumor site, No. (%)	
Left colon or rectum	31 (73.8)
Right colon	10 (23.8)
Unknown	1 (2.4)
Metastatic disease at diagnosis, No. (%)	
Yes	16 (38.1)
No	26 (61.9)
Liver metastasis, No. (%)	
Yes	25 (59.5)
No	16 (38.1)
Unknown	1 (2.4)
Metastatic lesions, No. (%)	
Single	16 (38.1)
Multiple	25 (59.5)
Unknown	1 (2.4)
Metastatic organs, No. (%)	
Single	16 (38.1)
Multiple	26 (61.9)
Received prior bevacizumab, No. (%)	
Yes	31 (73.8)
No	10 (23.8)
Unknown	1 (2.4)
RAS mutation, No. (%)	
KRAS	38 (90.5)
NRAS	1 (2.4)
Unspecified	3 (7.1)

Abbreviation: ECOG PS, Eastern Cooperative Oncology Group performance status.

### 2.2 Tumor Response

To assess tumor response, among 40 evaluable patients, the best overall response included two CRs (5.0%), 19 PRs (47.5%), 18 cases of SD (45.0%), and one PD (2.5%). The ORR was 52.5% (95% CI: 36.13, 68.49), and the DCR was 97.5% (95% CI: 86.84, 99.94) (Table 2). As detailed in Figure 2A, which illustrates individual treatment exposure and response duration, the majority of patients achieving CR or PR experienced durable responses, with many exceeding 6 months on treatment. Treatment discontinuation was primarily due to disease progression, while a minority withdrew owing to adverse events. Further analysis of tumor shrinkage is presented in Figure 2B. The waterfall plot demonstrates that 21 patients (52.5%) achieved tumor shrinkage greater than the 30% threshold for a PR, aligning with the ORR. The degree of reduction varied widely, with a maximum reduction exceeding 80% and a median best change of −41.5%. The single case of PD exhibited tumor growth greater than 20%. In summary, the regimen demonstrated substantial antitumor activity.

**TABLE 2:** Summary of tumor response and survival benefit.

Best overall response, *n* (%)	All patients, (*n* = 40)^a^
CR	2 (5.0)
PR	19 (47.5)
SD	18 (45.0)
PD	1 (2.5)
ORR, *n* (%)	21 (52.5)
DCR, *n* (%)	39 (97.5)
Median follow‐up, months (95% CI)	8.31 (6.05, 10.87)
PFS, median (95% CI), months	8.08 (6.18, 9.46)
3‐month PFS rate	85.27 (70.10, 93.10)
6‐month PFS rate	73.53 (56.05, 84.93)
9‐month PFS rate	35.92 (17.89, 54.39)
12‐month PFS rate	15.4 (1.49, 43.49)
OS, median (95% CI), months	NR

Abbreviations: CI, confidence interval; CR, complete response; NR, not reached; OS, overall survival; PD, progressive disease; PFS, progression‐free survival; PR, partial response; SD, stable disease.

^a^ Two patients were not evaluable for response (one withdrew consent, one had early progression before the first imaging assessment).

**FIGURE 2:**
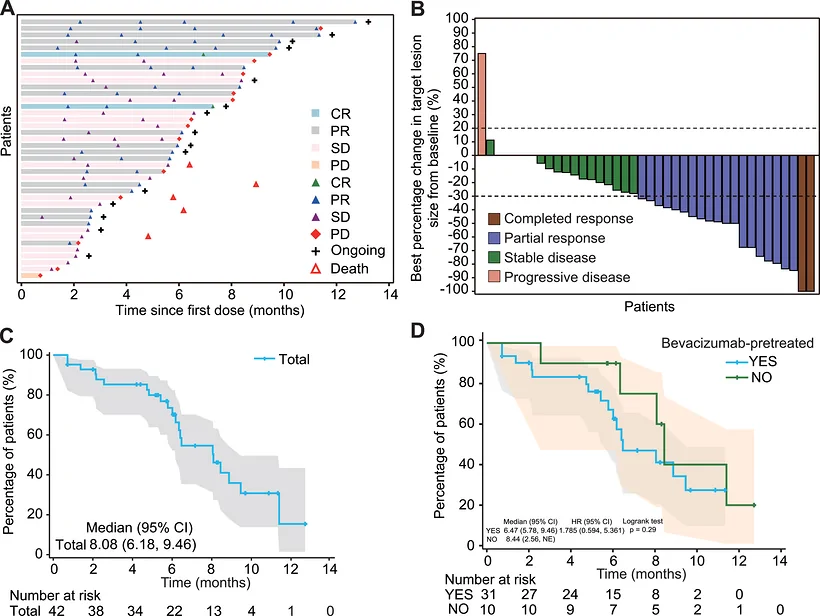
Clinical responses and survival of all patients. (A) Treatment exposure and response duration (*n* = 40 evaluable patients). The length of each bar represents the duration of treatment for each patient. The timeline starts from the treatment period. Tumor response was assessed according to RECIST v.1.1. (B) Best percentage change in the target lesion from baseline (*n* = 40 evaluable patients). The dashed line at 30% change represents the RECIST v.1.1 cutoff for a PR. (C) The PFS curve in total population. (D) PFS curves according to prior bevacizumab use. The corresponding hazard ratio (HR) with 95% confidence interval and log‐rank *p*‐value are shown. Tick marks indicate censored observations.

### 2.3 Progression‐Free Survival

To evaluate PFS, after a median follow‐up of 8.31 months, the median PFS reached 8.08 months (95% CI: 6.18, 9.46). The Kaplan–Meier PFS curve (Figure 2C) shows a steady decline beginning after approximately 6 months, consistent with the calculated median. The 3‐, 6‐, 9‐, and 12‐ month Kaplan–Meier PFS rates were 85.27%, 73.53%, 35.92%, and 15.4%, respectively (Table 2). Subgroup analyses of PFS were performed based on key baseline characteristics (Figure S1). Notably, patients with a single metastatic site showed a trend towards longer PFS compared to those with multiple sites. Regarding prior bevacizumab exposure, no statistically significant difference in PFS was observed, although a numerical trend favoring patients without prior bevacizumab was noted (median PFS 8.44 vs. 6.47 months; HR = 1.785, 95% CI: 0.594, 5.361) (Figure 2D). In summary, the median PFS was 8.08 months, and subgroup analyses showed no statistically significant differences based on prior bevacizumab use or other baseline characteristics, with a numerical trend favoring patients without prior bevacizumab.

### 2.4 Rate of Conversion to Surgery

To determine the rate of conversion to surgery, in this study, three patients (7.14%) with initially unresectable disease met the criteria for conversion surgery after receiving combination therapy, were subsequently withdrawn from the study, and underwent curative surgical resection. Table S1 summarizes their baseline characteristics and clinical course surrounding the surgical intervention. These patients, all male (aged 48, 57, and 68), had liver and/or lung metastases. Following the study regimen, all achieved partial response. The significant tumor shrinkage observed enabled curative‐intent surgical resection after 4–6 months of combination therapy. Figure 3 presents the pre‐ and post‐treatment CT images for 1 patient who underwent conversion surgery. This successful conversion highlights the potential of this regimen to create a window of surgical opportunity for a portion of patients with unresectable disease at baseline.

**FIGURE 3:**
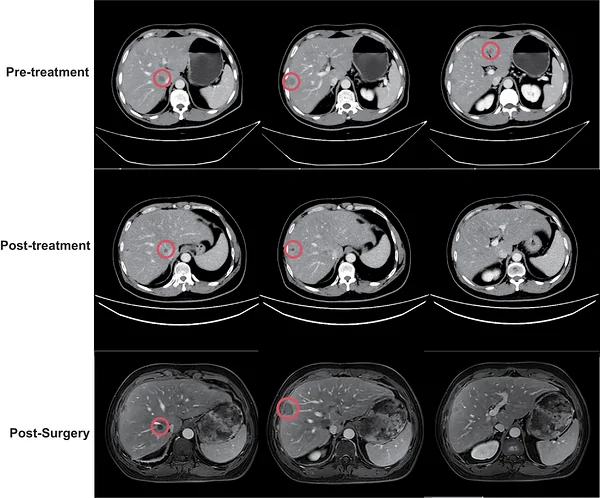
CT images of 1 patient before and after treatment. Red circles indicate three independent liver metastases at baseline (top), after fruquintinib plus chemotherapy, and after surgery (bottom). All three lesions shrank after treatment, one became undetectable, and all were subsequently resected.

### 2.5 Subgroup Analyses of PFS

To evaluate PFS across various baseline characteristics, subgroup analyses were conducted. PFS was not significantly different when comparing subgroups defined by gender, age groups, primary tumor location, presence of liver metastasis, number of previous bevacizumab exposure, number of metastatic sites, liver metastasis, or ECOG performance status (Figure 4). These exploratory analyses suggest consistent treatment effects across subgroups.

**FIGURE 4:**
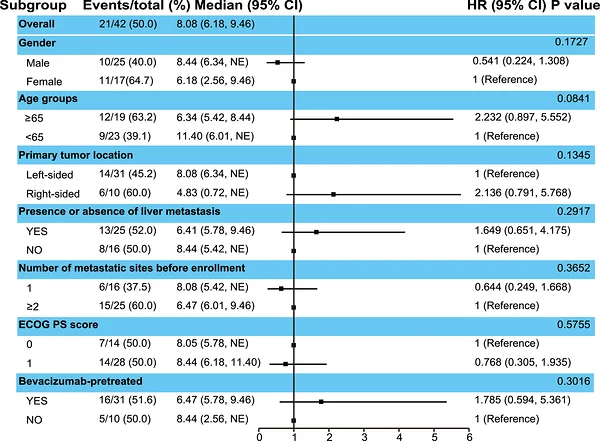
Subgroup analyses of progression‐free survival.

### 2.6 Safety and Feasibility

Safety and feasibility were assessed in all 42 patients. Table 3 summarizes the treatment‐related adverse events (TRAEs). In total, 27 patients (64.3%) experienced at least one TRAE, corresponding to 90 events. Grade ≥ 3 AEs were observed in 3 patients (7.1%), representing 8 events. The most common AEs of any grade were hypertension (23.8%), diarrhea (19.0%), and mucositis (19.0%). Grade ≥ 3 AEs included diarrhea (4.8%), thrombocytopenia (2.4%), leukopenia (2.4%), fever (2.4%), and urinary tract infection (2.4%) (Table 3). No previously unrecognized safety signals emerged, and the observed AE pattern matched with VEGFR inhibitors. All of these complications were alleviated after symptom‐mitigating treatment, no fatalities due to treatment were recorded. Overall, the safety profile was manageable, with no new signals identified.

**TABLE 3:** Treatment‐related adverse events (TRAEs) in 42 participants.

	Any grade		Grade ≥ 3	
Treatment‐related adverse events	Patients (%)	Events	Patients (%)	Events
All TRAEs	27 (64.3)	90	3 (7.1)	8
**Hematologic TRAEs**	5 (11.9)	9	1 (2.4)	4
Thrombocytopenia	4 (9.5)	5	1 (2.4)	2
Leukopenia	3 (7.1)	4	1 (2.4)	2
Neutropenia	0 (0.0)	0	0 (0.0)	0
Anemia	0 (0.0)	0	0 (0.0)	0
**Non‐hematologic TRAEs**	26 (61.9)	81	3 (7.1)	4
Hypertension	10 (23.8)	11	0 (0.0)	0
Diarrhea	8 (19.0)	10	2 (4.8)	2
Mucositis	8 (19.0)	8	0 (0.0)	0
Hand‐foot syndrome	5 (11.9)	9	0 (0.0)	0
Bleeding	4 (9.5)	5	0 (0.0)	0
Gastrointestinal adverse reaction	4 (9.5)	5	0 (0.0)	0
Abdominal pain	4 (9.5)	4	0 (0.0)	0
Fatigue	4 (9.5)	4	0 (0.0)	0
Hoarseness	4 (9.5)	4	0 (0.0)	0
Pain	3 (7.1)	3	0 (0.0)	0
Fever	2 (4.8)	3	1 (2.4)	1
Constipation	2 (4.8)	2	0 (0.0)	0
Decreased appetite	1 (2.4)	3	0 (0.0)	0
Alopecia	1 (2.4)	1	0 (0.0)	0
Dark urine	1 (2.4)	1	0 (0.0)	0
Edema	2 (4.8)	3	0 (0.0)	0
Elevated blood glucose	1 (2.4)	1	0 (0.0)	0
Headache	1 (2.4)	1	0 (0.0)	0
Hiccups	1 (2.4)	1	0 (0.0)	0
Proteinuria	1 (2.4)	1	0 (0.0)	0
Urinary tract infection	1 (2.4)	1	1 (2.4)	1

Dose modifications were required in a minority of patients. Fruquintinib dose was reduced in 4 patients (9.5%), most commonly due to hand‐foot syndrome (3 cases), and temporarily interrupted in 6 patients (14.3%), most commonly due to hypertension or thrombocytopenia (2 cases each). Chemotherapy dose was reduced in 1 patient (2.4%) due to decreased appetite, and chemotherapy was delayed or interrupted in 5 patients (11.9%), including one cycle extension due to fatigue. Patient‐level data are detailed in Table S2.

## 3 Discussion

This multicenter, single‐arm phase II trial demonstrates that fruquintinib combined with FOLFIRI/FOLFOX chemotherapy yields considerable efficacy as a second‐line treatment for patients with RAS‐mutant mCRC, achieving a median PFS of 8.08 months. This PFS outcome ranks among the longest reported for these patients in the second‐line setting, exceeding the overall median PFS of approximately 5–7 months observed in pivotal bevacizumab‐ or ramucirumab‐based trials such as ML18147, RAISE, BEBYP, and TRUSTY. Although these trials did not report median PFS specifically for the RAS‐mutant subgroup, subgroup analyses showed consistent benefit (ML18147: HR 0.70, 95% CI: 0.56, 0.89; RAISE: HR 0.88, 95% CI: 0.69, 1.004) [12, 13, 14, 26]. Notably, while these historical trials predominantly enrolled bevacizumab‐pretreated patients, 73.8% of patients in our study also had prior bevacizumab exposure, yet still achieved a median PFS of 8.08 months. However, cross‐trial comparisons should be interpreted with caution due to differences in study design, patient selection, and treatment eras. Our findings are hypothesis‐generating and require confirmation in a randomized controlled trial. The regimen also elicited a high ORR of 52.5% and a DCR of 97.5%. Notably, this study reports a conversion surgery rate of 7.14% (3/42) in RAS‐mutant mCRC patients receiving second‐line therapy–a result that surpasses historical second‐line benchmarks (0.75%–4.5%) [13, 26, 27] and approaches rates observed in first‐line settings (12%–14%) [28]. These findings highlight the ability of this fruquintinib‐containing regimen both to prolong PFS and to meaningfully enable curative‐intent resection. This efficacy may stem from fruquintinib's high selectivity and oral bioavailability, which promote sustained VEGFR inhibition and may overcome resistance to prior anti‐angiogenic therapy, thus underscoring the persistent value of VEGF pathway blockade in RAS‐mutant disease [23].

These results reinforce and extend the established role of VEGF pathway inhibition in RAS‐mutant mCRC following first‐line progression, even though cross‐study analyses suggest a diminished benefit in the second‐line treatment following previous bevacizumab exposure. Whether continuing bevacizumab (as in BEBYP, ML18147, and TRUSTY) [12, 13, 14] or switching to another agent such as ramucirumab [26], the median PFS in these bevacizumab‐pretreated cohorts is consistently shorter than in populations without such prior therapy. This pattern suggests that tumors may develop resistance to VEGF‐pathway inhibition [29], limiting the efficacy of subsequent anti‐angiogenic strategies and underscoring a significant unmet need in this patient population. Notably, in the ONSEMBLE trial, despite the addition of onvansertib, the median PFS was only 6.6 months in bevacizumab‐exposed patients, compared with 14.9 months in bevacizumab‐naive patients [30]. This further supports the concept that prior bevacizumab exposure may limit the efficacy of subsequent therapies, including novel targeted combinations.

In contrast, no statistically significant difference in PFS was observed based on prior bevacizumab use, although a numerical trend favoring patients without prior bevacizumab was noted (median PFS 8.44 vs. 6.47 months; HR = 1.785, 95% CI: 0.594, 5.361). Given the small sample size, this subgroup finding should be considered exploratory and requires validation in randomized trials. These data should be interpreted as hypothesis‐generating only, and the numerical difference should not be overinterpreted. This consistent performance underscores a potential distinct advantage for fruquintinib‐based therapy specifically in patients previously treated with bevacizumab. The superior PFS and notably higher conversion surgery rate observed in our study may be attributed to fruquintinib's potent specificity for VEGFR‐1, ‐2, and ‐3 and its oral bioavailability, which together could provide more sustained target inhibition and potentially greater tumor vasculature normalization than prior agents [20]. This mechanism may explain the enhanced antitumor activity, particularly in a population enriched with prior bevacizumab exposure, and is further substantiated by our subgroup analysis showing consistent efficacy of fruquintinib regardless of prior anti‐angiogenic treatment. Due to the very small number of patients in the FOLFOX group (*n* = 3), subgroup efficacy comparison by chemotherapy backbone was not performed.

While targeted agents such as KRAS G12C‐specific inhibitors [31] and onvansertib (a PLK1 inhibitor) [30] show activity only in molecularly defined subgroups and face limitations in applicability and resistance, our fruquintinib‐based regimen offers a broadly applicable “wide‐spectrum” strategy for all RAS‐mutant patients. The pronounced efficacy observed, including deep responses and a notable conversion surgery rate, can be attributed to a synergistic mechanism [25]: fruquintinib, as a highly selective oral VEGFR inhibitor, may promote tumor vascular normalization and improve the tumor microenvironment, thereby enhancing chemotherapeutic delivery and overcoming resistance [23]. The conversion surgeries provide compelling clinical evidence supporting this synergistic effect. This rationale aligns with evidence from other multi‐pathway modulation approaches, such as chemo‐bevacizumab plus anti‐PD‐1 in MSS mCRC (69.2% RAS‐mutant), which yielded a median PFS of 8.27 months and exhibited a marked tendency toward better PFS for the RAS‐mutant subgroup (HR = 0.57, *p* = 0.068) [32]. Collectively, these findings highlight fruquintinib combined with chemotherapy as a promising, mechanistically grounded second‐line option capable of delivering meaningful clinical benefit in this challenging, anti‐angiogenic‐exposed population.

As expected, the toxicity patterns of the individual agents were reflected in the manageable safety profile of the combination, with no new safety signals emerging [22]. Among 42 patients, TRAEs of any grade occurred in 27 (64.3%), and grade 3/4 AEs were observed in only 3 (7.1%). The most common AEs included hypertension (23.8%, all Grade 1–2), diarrhea (19.0%, with 4.8% Grade 3–4), and mucositis (19.0%, all Grade 1–2), all of which were manageable through supportive measures. This safety profile compares favorably with other second‐line targeted combinations. For example, in the phase II trial testing onvansertib combined with FOLFIRI/bevacizumab, 69.8% of patients experienced grade 3/4 AEs, including neutropenia in 35.8% [30]. Similarly, while angiogenesis inhibitors including bevacizumab, aflibercept, and ramucirumab confer well‐characterized risks (e.g., hypertension, bleeding, proteinuria) [13, 26, 33], severe AEs occurred noticeably less frequently in our study, supporting the tolerability of fruquintinib in combination with chemotherapy. This favorable risk‐benefit balance, which characterized by a low rate of severe toxicities alongside significant efficacy, underscores the clinical viability of this regimen as a promising second‐line option in the treatment of RAS‐mutant mCRC.

Several limitations of this study warrant acknowledgment, including its single‐arm (non‐randomized) design, limited sample size, and the consequent inability to fully account for potential selection bias and unmeasured confounding variables. The absence of dynamic biomarker monitoring also limits the exploration of predictive factors and the mechanistic understanding of how TKI‐based therapy retains activity after prior bevacizumab exposure—a question that warrants dedicated translational investigation. In addition, the follow‐up duration is still limited, and extended follow‐up is required to assess long‐term outcomes (e.g., OS). Despite these limitations, the robust efficacy signals justify further investigation. A definitive randomized phase III trial evaluating fruquintinib plus chemotherapy versus standard second‐line regimens is warranted to confirm these findings. Future research should also explore this combination in earlier treatment settings (e.g., first‐line or maintenance therapy) and to investigate biomarkers predictive of patient benefit, particularly those with potential for conversion therapy.

## 4 Conclusion

Fruquintinib combined with FOLFIRI/FOLFOX demonstrates encouraging effectiveness and an acceptable tolerability profile in patients with RAS‐mutant mCRC. Distinguished by a high response rate that is independent of prior bevacizumab use, this regimen represents a particularly promising second‐line option compared to other emerging strategies. The profound responses observed, including successful conversion surgery, offering the potential for altered disease trajectory and even cure in a subset of this poor‐prognosis population.

## 5 Materials and Methods

### 5.1 Study Design and Participants

This is a multicenter, open‐label, single‐arm phase II clinical trial that assesses the effectiveness and tolerability of fruquintinib (also known as HMPL‐013) in combination with FOLFIRI or FOLFOX chemotherapy in patients with RAS‐mutant mCRC refractory to first‐line therapy. The trial is being carried out at four centers in China: Fudan University Shanghai Cancer Center, Shanghai Minhang Cancer Center, Ruijin Hospital, and Baoshan Branch of Renji Hospital. The study was approved by the independent ethics boards of all participating institutions, and written informed consent was obtained from every patient prior to enrollment.

Patients must be 18 years of age or older and have histologically or cytologically confirmed unresectable metastatic colorectal adenocarcinoma, confirmed RAS mutation, and evidence of disease progression following prior exposure to fluoropyrimidine‐, oxaliplatin‐, or irinotecan‐containing regimens. Patients were considered eligible if they had at least one measurable lesion per RECIST v1.1, an ECOG performance status of 0 or 1, and a life expectancy of ≥ 12 weeks. In addition, adequate bone marrow reserve (neutrophils ≥ 1.5×10^9^/L, platelets ≥ 100×10^9^/L, hemoglobin ≥ 9 g/dL), hepatic function (AST/ALT ≤ 2.5 × ULN, ≤ 5 × ULN in the presence of liver metastases; total bilirubin ≤ 1.5 × ULN), renal function (serum creatinine ≤ 1.5 × ULN or creatinine clearance ≥ 60 mL/min), and normal coagulation parameters were mandatory. Key exclusion criteria include prior treatment with small‐molecule VEGFR inhibitors (e.g., fruquintinib, regorafenib), symptomatic brain metastases, uncontrolled hypertension (≥ 140/90 mmHg despite medication), coagulation disorders or active bleeding, significant cardiac disease, other malignancies within 5 years, and known hypersensitivity to the study drugs.

### 5.2 Treatment and Assessments

Patients are given fruquintinib at an oral dose of 4 mg once daily for Days 1–21 of every 28‐day cycle, together with either FOLFIRI or FOLFOX chemotherapy based on prior treatment history. The FOLFIRI chemotherapy consists of three drugs given on Days 1 and 15: irinotecan (180 mg/m^2^ IV over 30–90 min), leucovorin (400 mg/m^2^ IV over 2 h), and 5‐fluorouracil (400 mg/m^2^ IV bolus followed by 1200 mg/m^2^/day continuous infusion for 46–48 h, total 2400 mg/m^2^ per cycle). The FOLFOX regimen includes oxaliplatin 85 mg/m^2^ IV over 2 h on Days 1 and 15, leucovorin 400 mg/m^2^ IV over 2 h on Days 1 and 15, and 5‐FU with the same dosing and schedule as in FOLFIRI.

Treatment is administered up to the point of disease progression, intolerable toxicity, patient withdrawal of consent, or a decision by the investigator. Dose modifications are permitted based on toxicity graded per NCI CTCAE v5.0. For fruquintinib, dose reductions (in 1 mg decrements, minimum 2 mg/day) or interruptions are allowed for managing adverse events. Specific guidelines are provided for managing hypertension, proteinuria, hepatic dysfunction, thrombocytopenia, bleeding, and hand‐foot skin reaction. Chemotherapy dose adjustments follow standard protocols for managing hematologic and non‐hematologic toxicities.

Tumor evaluations were carried out every 8 weeks by means of CT or MRI according to RECIST v1.1. Safety monitoring encompassed surveillance of adverse events, laboratory parameters, vital signs, ECG, and echocardiography.

### 5.3 Outcomes

The primary endpoint was PFS, calculated from the start of treatment until either disease progression or death (any cause), whichever happened first. Secondary endpoints comprised ORR, DCR, OS, and safety. ORR was defined as the percentage of patients with either CR or PR; DCR encompassed CR, PR, and SD maintained for ≥ 6 weeks. Safety was evaluated based on the frequency and grade of adverse events, lab test abnormalities, and changes in vital signs and cardiac function.

### 5.4 Statistical Analysis

The sample size calculation is based on the assumption that fruquintinib plus chemotherapy will improve median PFS from 4.1 months to 6.0 months. The historical control median PFS of 4.1 months was derived from the chemotherapy alone arm of the ML18147 trial, in which patients with mCRC received second‐line chemotherapy without bevacizumab after progression on first‐line bevacizumab‐containing treatment (median PFS 4.1 months, 95% CI: 3.7, 4.4) [13]. This estimate serves as a conservative benchmark for the RAS‐mutant population, for which historical data are limited. Under this assumption, with a one‐sided significance level of 0.1, 80% power, an enrollment period of 24 months, and an additional 12 months of follow‐up, a total of 38 evaluable patients are required. Accounting for a 10% dropout rate, the planned sample size is 43 patients.

Efficacy was evaluated using two populations: the full analysis set (FAS), comprising all patients who received at least one study drug dose (intent‐to‐treat principle), and the per‐protocol set (PPS), consisting of those who adhered to the protocol. The safety analysis population encompassed all patients who had taken at least one dose and had post‐baseline safety data available.

The Kaplan–Meier method was applied to estimate PFS and OS, and the corresponding median values along with 95% confidence intervals were reported. For ORR and DCR, proportions with 95% CIs were calculated. Descriptive statistics summarized the safety data, including the frequency and severity of adverse events classified according to NCI CTCAE v5.0. All statistical evaluations were carried out using SAS (version 9.2), applying a two‐sided significance level of 0.05 except where otherwise noted.

All subgroup analyses (e.g., by prior bevacizumab use, number of metastatic sites) were pre‐specified as exploratory. No adjustment for multiple comparisons was performed. Results should be interpreted as hypothesis‐generating.

## Author Contributions

Y. X. and M.T. initiated and planned this study and had full access to all data. Y.X., C.L., Y.D., X.J., Z.C., X.Z. and Y.X. recruited and treated patients. Y.X., C.L. and W.Z. collected data. Y.X. and C.L. performed statistical analyses. Y.X. and C.L. wrote the initial version, which was then reviewed and revised by all authors. All authors have read and approved the final manuscript and assume responsibility for its contents.

## Funding

This work was supported by Natural Science Foundation of Shanghai (24ZR1413400) and National Natural Science Foundation of China (no. 82573916, no. 82573404, and no. 82606038).

## Ethics Statement

This study was carried out following the Declaration of Helsinki and the principles of Good Clinical Practice, and received approval from the ethics committee of Fudan University Shanghai Cancer Center (Approval No. 2209261‐16‐2405D). All participants provided written informed consent before treatment, and this trial adhered to all relevant ethical considerations. This trial has been registered in ClinicalTrials.gov (NCT05634590).

## Conflicts of Interest

The authors declare no conflicts of interest.

## Supporting information




**Supporting File 01**: mco271137‐sup‐0001‐SuppMatData.docx

## Data Availability

The data that support the findings of this study can be obtained from the corresponding author, Ye Xu (yexu@shmu.edu.cn), upon reasonable request.
