# Supplementary material for: Fruquintinib Plus FOLFIRI/FOLFOX as Second‐Line Therapy in Patients With RAS‐Mutant Metastatic Colorectal Cancer: A Multicenter, Open‐Label, Single‐Arm Phase II Study
Source: MedComm (2020). 2026 Nov 8;7(11):e71137. doi: 10.1002/mco2.71137 (PMC13745995; doi:10.1002/mco2.71137)
Supplement: Supplementary file 1 [file MCO2-7-e71137-s001.docx]

**Fruquintinib plus FOLFIRI/FOLFOX as Second-line Therapy in Patients with RAS-mutant Metastatic Colorectal Cancer: A Multicenter, Open-label, Single-arm Phase II Study**

**Yun Xu^1,2,#^, Cong Li^1,2,#^, Yiwei Zeng^1,2,#^, Yuchen Wu^1,2^, Yuedi Dai^3^, Xiaopin Ji^4^, Zhe Cui^5^, Xiaodong Zhu^1,2^, Ming Tang^6,*^, Ye Xu^1,2,*^**

^1^Department of Colorectal Surgery, Fudan University Shanghai Cancer Center, Shanghai 200032, China

^2^Department of Oncology, Shanghai Medical College, Fudan University, Shanghai 200000, China

^3^Department of Oncology, Shanghai Minhang Cancer Center, Shanghai 201199, China

^4^Ruijin Hospital, Shanghai Jiao Tong University School of Medicine, Shanghai 200020, China

^5^Baoshan Branch of Renji Hospital Affiliated to Shanghai Jiao Tong University School of Medicine, Shanghai 200444, China

^6^Shanghai Key Laboratory of Maternal Fetal Medicine, Shanghai Institute of Maternal-Fetal Medicine and Gynecologic Oncology, Clinical and Translational Research Center, Shanghai First Maternity and Infant Hospital, School of Medicine, Tongji University, Shanghai 201204, China

^#^Yun Xu, Cong Li and Yiwei Zeng contributed equally to this work.

*Correspondence: Ming Tang [(tangming@bjmu.edu.cn);](mailto:(tangming@bjmu.edu.cn);) Ye Xu [(yexu@shmu.edu.cn)](mailto:(yexu@shmu.edu.cn))

Figure S1. Kaplan-Meier curves for progression-free survival by baseline characteristics.

Stratified by (A) gender, (B) age (<65 vs. ≥65 years), (C) primary tumor location (left-sided vs. right-sided), (D) liver metastasis (yes vs. no), (E) number of metastatic sites (single vs. multiple), (F) ECOG performance status (0 vs. 1), and (G) prior bevacizumab use (yes vs. no).

| **Table S1. Clinical characteristics and detailed treatment regimens of the 3 patients who received conversion surgery after treatment.** | | | |
| --- | --- | --- | --- |
| Patients | 1 | 2 | 3 |
| Gender | Male | Male | Male |
| Age | 48 | 57 | 68 |
| Metastasis organ | Lung and Liver | Liver | Liver |
| Prior bevacizumab | Yes | No | Yes |
| Prior Surgery | Anterior resection of the rectum | No | No |
| BOR | PR | PR | PR |
| Surgery | Right hepatectomy combined with intraoperative radiofrequency ablation | Laparoscopic low anterior resection (Dixon's procedure) and Liver ablation therapy | Laparoscopic low anterior resection (Dixon's procedure) |
| Abbreviation: BOR, best overall response. | | | |

| **Table S2. Detailed treatment-related adverse events (TRAEs) and dose modifications per patient** | | | | |
| --- | --- | --- | --- | --- |
| **ID** | **Name Initials** | **AE Preferred Term** | **AE Grade** | **Intervention** |
| 0101 | DJZH | Edema | Grade 1 |  |
|  |  | Edema | Grade 1 |  |
|  |  |  |  |  |
| 0102 | SXTU | Hypertension | Grade 2 | Fruquintinib: antihypertensive treatment |
|  |  | Hand–foot syndrome | Grade 2 | None |
|  |  | Hand–foot syndrome | Grade 2 | Fruquintinib: Dose reduction to 3 mg from August 19 as recommended by the physician |
|  |  |  |  |  |
| 0103 | QHHI | Hand–foot syndrome | Grade 2 | Fruquintinib: Dose reduction to 3 mg from August 6 |
|  |  | Diarrhea | Grade 1 | Fruquintinib: Smecta was recommended |
|  |  | Fatigue | Grade 1 | None |
|  |  | Hand–foot syndrome | Grade 1 | Fruquintinib: interrupted from 2023-08-29; symptoms improved; resumed on 2023-09-13 with dose reduction to 2 mg. |
|  |  |  |  |  |
| 0104 | ZXCH | Hypertension | Grade 1 | Fruquintinib: Antihypertensive treatment |
|  |  | Hypertension | Grade 1 | Fruquintinib: Dose reduction to 3 mg from August 8 |
|  |  | Leukopenia | Grade 2 | Leukocyte-elevating injection administered |
|  |  | Constipation | Grade 1 | Glycerin enema and probiotics |
|  |  | Gastrointestinal adverse reaction | Grade 1 |  |
|  |  |  |  |  |
| 0105 | XXPI | Hypertension | Grade 1 |  |
|  |  | Diarrhea | Grade 2 |  |
|  |  | Mucositis | Grade 2 |  |
|  |  | Hand–foot syndrome | Grade 2 |  |
|  |  | Bleeding | Grade 1 |  |
|  |  | Hand–foot syndrome | Grade 2 |  |
|  |  | Pain | Grade 2 |  |
|  |  | Edema | Grade 2 |  |
|  |  | Hand–foot syndrome | Grade 2 |  |
|  |  | Urinary tract infection | Grade 3 |  |
|  |  | Bleeding | Grade 2 |  |
|  |  |  |  |  |
| 0106 | ZHLA | Thrombocytopenia | Grade 2 |  |
|  |  | Abdominal pain | Grade 2 |  |
|  |  |  |  |  |
| 0108 | CYQI | Fatigue | Grade 1 |  |
|  |  | Headache | Grade 1 |  |
|  |  | Gastrointestinal adverse reaction | Grade 1 |  |
|  |  | Mucositis | Grade 1 |  |
|  |  | Hypertension | Grade 1 |  |
|  |  |  |  |  |
| 0109 | LHHO |  |  |  |
|  |  |  |  |  |
| 0110 | WAHU | Abdominal pain | Grade 1 |  |
|  |  | Fatigue | Grade 2 |  |
|  |  | Mucositis | Grade 1 |  |
|  |  |  |  |  |
| 0111 | DYXI | Hypertension | Grade 1 |  |
|  |  | Hoarseness | Grade 1 |  |
|  |  | Abdominal pain | Grade 1 |  |
|  |  | Mucositis | Grade 1 |  |
|  |  |  |  |  |
| 0112 | DSLO | Dark urine | Grade 1 |  |
|  |  | Hoarseness | Grade 1 |  |
|  |  | Hand–foot syndrome | Grade 1 |  |
|  |  | Hypertension | Grade 1 |  |
|  |  | Bleeding | Grade 1 |  |
|  |  | Mucositis | Grade 1 |  |
|  |  |  |  |  |
| 0115 | CGQI | Hypertension | Grade 1 |  |
|  |  | Gastrointestinal adverse reaction | Grade 1 |  |
|  |  | Gastrointestinal adverse reaction | Grade 1 |  |
|  |  | Hoarseness | Grade 1 |  |
|  |  |  |  |  |
| 0116 | ZYZH | Diarrhea | Grade 2 |  |
|  |  | Fatigue | Grade 1 |  |
|  |  |  |  |  |
| 0117 | HAFA | Fever | Grade 1 |  |
|  |  | Diarrhea | Grade 2 |  |
|  |  |  |  |  |
| 0118 | ZJWE | Diarrhea | Grade 1 |  |
|  |  | Hand–foot syndrome | Grade 2 | Fruquintinib: Dose reduced to 3 mg starting 2024-05-31; 4 mg resumed on July 15 |
|  |  | Diarrhea | Grade 3 | Treatment interrupted from 2024-07-20; inpatient treatment provided. |
|  |  |  |  |  |
| 0119 | ZSYA | Hypertension | Grade 2 | Fruquintinib: interrupted on 2024-05-15 |
|  |  |  |  |  |
| 0120 | LIQI | Mucositis | Grade 1 | Fruquintinib: interrupted from 2024-05-20 to 2024-06-10 |
|  |  |  |  |  |
| 0121 | LYJU | Abdominal pain | Grade 1 |  |
|  |  | Gastrointestinal adverse reaction | Grade 1 |  |
|  |  |  |  |  |
| 0123 | XXYI | Diarrhea | Grade 2 |  |
|  |  | Mucositis | Grade 1 |  |
|  |  | Fever | Grade 1 |  |
|  |  | Thrombocytopenia | Grade 4 |  |
|  |  | Leukopenia | Grade 4 |  |
|  |  | Fever | Grade 4 |  |
|  |  | Diarrhea | Grade 4 |  |
|  |  | Leukopenia | Grade 4 |  |
|  |  | Thrombocytopenia | Grade 4 |  |
|  |  |  |  |  |
| 0124 | LSCH | Constipation | Grade 1 |  |
|  |  | Diarrhea | Grade 1 |  |
|  |  |  |  |  |
| 0125 | SRFA | Diarrhea | Grade 2 | Chemotherapy interrupted; fruquintinib monotherapy continued |
|  |  |  |  |  |
| 0126 | CHBI | Thrombocytopenia | Grade 2 | Fruquintinib + chemotherapy: Treatment interrupted from 2024-08-21 to 2024-08-30 |
|  |  | Leukopenia | Grade 2 |  |
|  |  |  |  |  |
| 0127 | ZJLI |  |  |  |
| 0130 | XIQI |  |  |  |
| 0131 | LJRO |  |  |  |
| 0132 | ZGQI |  |  |  |
| 0134 | YZGU |  |  |  |
|  |  |  |  |  |
| 0136 | ZHCU | Hypertension | Grade 1 |  |
|  |  | Thrombocytopenia | Grade 1 | Treatment interrupted on April 16 and resumed on May 22 |
|  |  |  |  |  |
| 0137 | ZPCI | Decreased appetite | Grade 1 | Intravenous chemotherapy interrupted |
|  |  | Decreased appetite | Grade 1 | Irinotecan dose reduction |
|  |  | Decreased appetite | Grade 1 |  |
|  |  |  |  |  |
| 0139 | DJBA | Pain | Grade 1 | Chemotherapy interrupted |
|  |  | Proteinuria | Grade 1 | Fruquintinib interrupted |
|  |  |  |  |  |
| 0141 | YJHU | Hoarseness | Grade 1 |  |
|  |  | Bleeding | Grade 1 |  |
|  |  | Mucositis | Grade 1 |  |
|  |  | Elevated blood glucose | Grade 1 |  |
|  |  |  |  |  |
| 0142 | ZXCH |  |  |  |
|  |  |  |  |  |
| 0143 | LHPO | Hiccups | Grade 1 |  |
|  |  | Bleeding | Grade 1 |  |
|  |  | Pain | Grade 1 |  |
|  |  | Alopecia | Grade 1 |  |
|  |  |  |  |  |
| 201 | SDRO |  |  |  |
| 0145 | LYCH | Hypertension | Grade 2 | Fruquintinib: Oral antihypertensive medication; cycle 2 treatment interrupted |
| 0147 | JCPI |  |  |  |
| 0148 | LAJU |  |  |  |
| 0149 | XIHO |  |  |  |
| 0150 | LUMI |  |  |  |
| 0151 | ZMMI |  |  |  |
| 0152 | JSCH |  |  |  |
| 0153 | MZLE |  |  |  |
| Note: Blank cells in the “Intervention” column indicate that no intervention was taken. | | | | |
